# Do we know more about the mechanobiology of the intervertebral disc in space than on Earth?

**DOI:** 10.1002/jsp2.70024

**Published:** 2025-02-18

**Authors:** Timothy Patrick Holsgrove, Isabelle Ebisch, Daniela Lazaro‐Pacheco

**Affiliations:** ^1^ Department of Engineering, Faculty of Environment, Science and Economy University of Exeter Exeter UK

**Keywords:** biomechanics, bioreactors, culture systems, intervertebral disc, mechanobiology, microgravity, physiological loading, space

## Abstract

This work provides a perspective on the loading protocols used in whole‐organ interverterbal disc culture studies using bioreactors. We put this in the context of in vivo spinal loading, and we put forward the case that the majority of previous bioreactor studies have more in common with spinal loading in space than on Earth. Finally, we provide an outlook for the future of bioreactor research, to provide data more relevant to spinal loading on Earth, and maximize the translational potential of findings to the clinical setting.

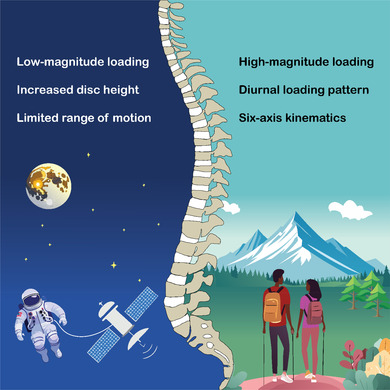

## 1 INTRODUCTION

It is well reported that low back pain is associated with huge direct and indirect treatment costs, losses in productivity, and years lived with disability.^1^, ^2^ While in many cases the exact source of pain is difficult to determine, it is understood that intervertebral disc (IVD) degeneration is a key issue, which may result in pain and disability. The degeneration of the IVD includes both mechanical and biological factors that are linked together in what has been described as a vicious circle,^3^ and studies have shown that the viability and gene expression of cells are influenced by the mechanical environment to which they are exposed.^4^ Various methods have been used to investigate IVD mechanobiology, ranging from 2D and 3D cell cultures, whole‐organ IVD culture studies, and small and large in vivo animal models. The culture of whole‐organ intervertebral discs (IVDs) in a bioreactor provides a valuable way to study the IVD as a whole, and the interaction between cells and the extracellular matrix under different conditions, including mechanical loading.

However, while mechanical loading has been shown to significantly and substantially influence cell viability and gene expression, we believe that the loading used in a large number of bioreactor studies has been closer to the loads that the IVD would be subjected to in a microgravity environment, rather than on Earth. We believe that this may limit the translation of findings to understand the progression of IVD degeneration on Earth, and the development and evaluation of treatments for it. Therefore, the aim of this perspective is to outline the development of bioreactors for the study of IVD mechanobiology, describe IVD loading on Earth and in a microgravity environment, highlight the differences between them, and compare them with a comprehensive review of loading regimes used in previous bioreactor studies, before providing recommendations for future research using IVD bioreactor systems.

## 2 BIOREACTORS FOR WHOLE‐ORGAN IVD CULTURE

Early bioreactors for IVD culture did not integrate mechanical loading and allowed the IVD specimens to freely swell during culture, or adjusted the osmotic pressure via the culture media to regulate the IVD swelling. Over the years, mechanical loading has gradually been incorporated into IVD bioreactors to enable a better representation of the in vivo environment and provide a greater understanding of IVD mechanobiology. This loading was first applied as static axial compression, then cyclic loading in axial compression, the application of load and recovery periods to replicate the diurnal cycle, and then the application of multi‐axis loading.^5^, ^6^

This progression has led to systems with the capability to provide a better understanding of IVD mechanobiology under more realistic conditions, improving our understanding of native disc physiology, and providing better methods for pre‐clinical testing. This also serves to bridge the gap between 2D and 3D cell culture models, which provide relatively low‐cost and high throughput but do not replicate physiological loading, and in vivo animal testing, which generally has a higher cost and lower throughput.^4^ By creating more physiological conditions in vitro, the use of bioreactors with mechanical loading can also work toward the principles of the 3Rs; to reduce, replace, and refine the use of animals in research.^7^

The gradual development of bioreactor designs to integrate diurnal or multi‐axis loading has suggested that these more physiological loading characteristics may create a more challenging environment for the cells of the IVD.^8^, ^9^ However, progress is still required to integrate mechanical loading that is truly physiological.^5^, ^6^ which would provide a greater ability to successfully translate bioreactor data to the human in vivo setting.

## 3 PHYSIOLOGICAL LOADING

With the integration of mechanical loading into whole‐organ IVD culture systems, many studies that have adopted cyclic loading and recovery periods have referred to “physiological loading,” to distinguish from unloaded control groups, or prior studies with groups subjected to static loading or free swelling conditions. However, not all loading is the same, and not all loading is physiological.

Intradiscal pressure studies conducted in vivo have provided valuable data about the intradiscal pressure in a small number of participants during different postures and activities such as lifting.^10^, ^11^ These studies have helped to understand the magnitude of positive pressure even during lying down (0.1–0.15 MPa), highlighting that although the IVD height and fluid content will fluctuate throughout the diurnal cycle, it is rarely, if ever, in a free‐swelling state. These studies have also shown the large variation in intradiscal pressure during different postures and activities, for example, the pressure of ~0.5 MPa during relaxed standing can increase more than four‐fold during flexion combined with lifting.^11^

Further data about spinal loading during activities of daily living (ADLs), albeit in participants that have undergone fusion surgery, have been obtained from instrumented vertebral body replacements, as part of the Orthoload database.^12^ Critically, this database provides the six‐axis load data, rather than the intradiscal pressure, and includes over 750 functional movements and ADLs for up to five participants. These data have demonstrated that even simple functional movements often involve six‐axis loads, and activities such as walking involve dynamic changes in forces and moments in all three planes. However, while this data helps to understand the complexity of loading, it may underestimate forces on the IVD in healthy individuals due to the vertebral body replacement being implanted in combination with posterior fixation, and the fusion procedure itself may also affect the way in which loads are transferred through the spine compared to healthy individuals and those that have not undergone spinal surgery.

Alternative methods to estimate spinal loading have been completed using in vivo motion capture combined with musculoskeletal modeling.^13^, ^14^ This provides the potential to estimate six‐axis spinal loads at multiple levels, in healthy participants, as well as specific patient populations, though the thorough validation of such models remains challenging. The use of this methodology in healthy participants, combined with in vitro testing, has shown that the six‐axis loads during functional movements such as flexion estimated from musculoskeletal modeling result in substantially different kinematics than the more simple pure moment loading protocols commonly used in biomechanical testing.^14^

While all of the above methods of measuring and estimating spinal loading have limitations, they demonstrate that in vivo loading on the spine is complex, that most activities involve a combination of changes in axial compression combined with both bending and shear loading, and therefore, may not be suitably replicated in vitro through the application of constant loads or simple waveforms in a limited number of axes. The above methods have also shown that physiological loading occurs over a range of rates, from quasistatic/low rates (<0.2 Hz) for activities such as sleeping and changing posture, to more dynamic loading (~1 Hz) associated with activities such as walking and running.

Finally, as whole‐organ IVD culture models are completed over multiple days, it is necessary to incorporate diurnal changes, such as a 16:8 h load: recovery protocol, with higher‐magnitude loading to replicate daily activities, and lower‐magnitude loading to replicate sleeping. However, while supine, prone, and lateral lying positions result in a relatively low intradiscal pressure of 0.1–0.15 MPa, changing posture in bed can lead to peak loads similar to those during daily activities (0.7–0.8 MPa),^11^ and sleep may also be disrupted by the need to use the bathroom, again leading to a short period of loading similar in magnitude to during the day. Similarly, it is likely that, for the majority of people, there will be periods of low‐magnitude loading during the day. Therefore, while it is important to replicate diurnal loading in bioreactor protocols, it may not be as simple as having a period of high‐magnitude loading followed by a period of low‐magnitude loading.

It is challenging to replicate the complex loads described above in vitro, but it is important that this challenge is recognized. While many previous IVD bioreactor studies have incorporated some characteristics of physiological loading, only a small number have applied loading in multiple axes,^8^, ^15^, ^16^, ^17^, ^18^, ^19^ and these studies, along with the much larger number of IVD bioreactor studies that have applied axial compression alone, have often been limited in terms of the replicating the complexity of physiological loading with respect to daily loading time, load magnitude, load rate, diurnal loading, or the recovery loading regime (Table 1).

**TABLE 1:** Many previous multi‐day IVD bioreactor studies have limited loading to axial compression, with a large proportion also adopting one or more of the following, which may limit translation of findings to in vivo loading on Earth: Static loading; low load rates; short loading periods each day; sub‐physiological loading magnitudes with respect to non‐sedentary activities of daily living; a zero load recovery regime; no replication of diurnal loading.

Author	Year	Axes	Loading regime	Loading (h/day)	Recovery regime
Šećerović et al^15^	2024	Axial compression Flexion/extension Axial rotation	0.1 MPa static axial compression ± 3°, ±6°, or 0–6° flexion/extension for ~10k cycles at 0.2 Hz ± 2°, ±4°, or 0–4° axial rotation ~10k cycles at 0.2 Hz	~1	Not reported
			0.1 MPa static axial compression ± 3°, ±6°, or 0–6° flexion/extension for ~100 k cycles at 1.0 Hz ± 2°, ±4°, or 0–4° axial rotation ~10k cycles at 1.0 Hz	~2	Not reported
Beatty et al^16^	2016	Axial compression Flexion/extension Lateral bending	5 min cycle comprising: 100 N of axial compression at 0.02 Hz; 2.2 Nm flexion at 0.04 Hz; 4.4 Nm extension at 0.04 Hz ± 1.9 Nm lateral bending at 0.04 Hz; and 150 s of zero load	16	Zero load
Frauchiger et al^17^	2018	Axial compression Axial rotation	0.2 MPa static axial compression ±2° axial rotation at 0.2 Hz	8	Zero load
		Axial compression	0.2 MPa static axial compression	8	Zero load
Chan et al^18^	2015	Axial compression Axial rotation	0.2 MPa static axial compression ± 2° axial rotation at 1.0 Hz	0, 1, 4, or 8	Zero load
Chan et al^8^	2013	Axial compression Axial rotation	0.2 MPa static axial compression ± 2° at 0.2 Hz	8	Zero load
		Axial compression Axial rotation	0.6 ± 0.2 MPa at 0.2 Hz ± 2° axial rotation at 0.2 Hz	8	Zero load
		Axial compression	0.6 ± 0.2 MPa at 0.2 Hz	8	Zero load
Chan et al^19^	2011	Axial compression Axial rotation	20 N static axial compression 0°, ±2°, ±5°, or ± 10° axial rotation at 0.1 Hz	1	20 N static axial compression
Zhou et al^30^	2024	Axial compression	0.02–0.2 MPa axial compression at 1 Hz 38% axial compression in 1 s on day 2/5/8/11/14/17/20/23/26/29	1	Zero load
			0.02–0.2 MPa axial compression at 1 Hz 38% axial compression in 1 s on day 2	1	Zero load
			0.02–0.2 MPa axial compression at 1 Hz	1	Zero load
Šećerović et al^26^	2022	Axial compression	0.02–0.2 MPa axial compression at 0.2 Hz	2	Zero load
Zhou et al^31^	2021	Axial compression	0.02–0.2 MPa axial compression at 0.2 Hz 50% axial compression in 1 s on day 2	2	Zero load
			0.02–0.2 MPa axial compression at 0.2 Hz	2	Zero load
Xing et al^32^	2020	Axial compression	0–0.5 MPa at 1 Hz	1.5	Zero load
Paul et al^33^	2018	Axial compression	0.09–0.11 MPa and 0.1–0.5 MPa at 1 Hz applied in alternating 30‐min periods	16	0.09–0.11 MPa at 1 Hz
Emanuel et al^28^	2018	Axial compression	50 ± 10 N and 150 ± 10 N at 1 Hz applied in alternating 30‐min periods	16	50 ± 10 N at 1 Hz
			50 ± 10 N and 300 ± 10 N at 1 Hz applied in alternating 30‐min periods	16	50 ± 10 N at 1 Hz
Navone et al^34^	2018	Axial compression	0.4–0.8 MPa at 10 Hz for two 4‐h periods separated by static 0.6 MPa	10	Static 0.2 MPa
Paul et al^35^	2017	Axial compression	~0.1 MPa and 0.1–0.5 MPa at 1 Hz applied in alternating 30‐min periods	16	~0.1 MPa at 1 Hz
			0.4–0.8 MPa at 1 Hz	16	~0.1 MPa 1 Hz
			Static 0.6 MPa	16	~0.1 MPa at 1 Hz
Chooi et al^36^	2016	Axial compression	Static 0.35 MPa	2	Zero load
			0.35 ± 0.25 MPa at 0.2 Hz	2	Zero load
Rosenzweig et al^37^	2016	Axial compression	2–7 days zero load; 2 days 0.1 MPa static axial compression; 0.1–0.3 MPa axial compression at 0.1 Hz in two 2‐h periods separated by 6 and 14 h	4	Static 0.1 MPa
			2–7 days zero load; 2 days 0.1 MPa static axial compression; 0.1–0.6 MPa axial compression at 0.1 Hz in two 2‐h periods separated by 6 and 14 h	4	Static 0.1 MPa
			2–7 days zero load; 2 days 0.1 MPa static axial compression; 0.1–1.2 MPa axial compression at 0.1 Hz in two 2‐h periods separated by 6 and 14 h	4	Static 0.1 MPa
Zhan et al^38^	2016	Axial compression	Static 0.5 MPa	24	n/a
Dudli et al^39^	2015	Axial compression	0.8–1.7 MPa at 1 Hz for 2500 cycles	~0.7	Zero load
Castro et al^40^	2014	Axial compression	150 ± 100 N at 1 Hz for 16 h with transition of 200 ± 100 N at 0.25 Hz for 1 h between active and recovery loading	16	50 ± 10 N at 1 Hz
Walter et al^41^	2014	Axial compression	Static 0.2 MPa	24	n/a
			Static 0.2 MPa	12	Static 0.1 MPa
			Static 0.6 MPa with two 5‐h periods of 0.6 ± 0.2 MPa at 0.1 Hz	16	Static 0.2 MPa
Paul et al^42^	2013	Axial compression	~0.1 MPa and 0.1–0.6 MPa at 1 Hz applied in alternating 30‐min periods	16	~0.1 MPa at 1 Hz
			~0.1 MPa and 0.4–0.8 MPa at 1 Hz applied in alternating 30‐min periods	16	~0.1 MPa at 1 Hz
			Static 0.6 MPa	16	~0.1 MPa at 1 Hz
Illien‐Jünger et al^43^	2012	Axial compression	Static 0.6 MPa with two 4‐h periods of 0.6 ± 0.2 MPa at 0.2 Hz	16	Static 0.2 MPa
			Static 0.6 MPa with two 4‐h periods of 0.6 ± 0.2 MPa at 10 Hz	16	Static 0.2 MPa
Paul et al^44^	2012	Axial compression	0.1–0.2 MPa at 1 Hz	24	n/a
			0.1–0.2 MPa and 0.1–0.6 MPa at 1 Hz applied in alternating 30‐min periods	16	0.1–0.2 MPa at 1 Hz
Haglund et al^45^	2011	Axial compression	7 days zero load; 2 days static 0.1 MPa; 0.1–0.3 MPa at 0.1 Hz in two 2‐h periods separated by 6 and 14 h recovery	4	Static 0.1 MPa
			7 days zero load; 2 days static 0.1 MPa; 0.1–0.6 MPa at 0.1 Hz in two 2‐h periods separated by 6 and 14 h recovery	4	Static 0.1 MPa
			7 days zero load; 2 days static 0.1 MPa; 0.1–1.2 MPa at 0.1 Hz in two 2‐h periods separated by 6 and 14 hours recovery	4	Static 0.1 MPa
Illien‐Jünger et al^29^	2010	Axial compression	Static 0.6 MPa with two 4‐h periods of 0.6 ± 0.2 MPa at 0.2 Hz	16	Static 0.2 MPa
			Static 0.6 MPa with two 4‐hour periods of 0.6 ± 0.2 MPa at 10 Hz	16	Static 0.2 MPa
Jünger et al^46^	2009	Axial compression	Static 0.6 MPa with two 4‐h periods of 0.6 ± 0.2 MPa at 0.2 Hz	16	Static 0.2 MPa
Korecki et al^47^	2008	Axial compression	0.2–0.4 MPa at 1 Hz for 1 min; 0.2–1.0 MPa at 1 Hz for 1 h; 0.2–0.4 MPa at 1 Hz for 1 min	~1	Static 0.2 MPa
Korecki et al^48^	2008	Axial compression	0.2–0.4 MPa at 1 Hz for 1 min	~0	Static 0.2 MPa
			0.2–0.4 MPa at 1 Hz for 1 min; 0.2–1.0 MPa at 1 Hz for 1 h; 0.2–0.4 MPa at 1 Hz for 1 min	~1	Static 0.2 MPa
			0.2–0.4 MPa at 1 Hz for 1 min; 0.2–2.5 MPa at 1 Hz for 1 h; 0.2–0.4 MPa at 1 Hz for 1 min	~1	Static 0.2 MPa
Korecki et al^9^	2007	Axial compression	Static 0.2 MPa	24	n/a
			Static 0.3 MPa	12	Static 0.1 MPa
Gantenbein et al^49^	2006	Axial compression	Static 0.2 MPa	24	n/a
			Static 0.8 MPa	16	Static 0.2 MPa
Lee at al^50^	2006	Axial compression	Static 5 kg (~0.25 MPa)	24	n/a
			Static 5 kg (~0.25 MPa) and static 20 kg (~1.0 MPa) for the last 6 h of culture	24	n/a
Ariga et al^51^	2003	Axial compression	Static 0.0 , 0.2, 0.4, 0.8, or 1.0 MPa	24	n/a

*Note*: Zero loaded (free‐swelling) or day 0 control groups that may have been included in the above studies have been omitted for brevity. Many studies do not report or depict the waveform used for non‐static loading or recovery regimes. All studies that do have used sine or triangle waves.

## 4 SPINAL LOADING IN SPACE

Though muscle and ligamentous structures are likely to prevent the in vivo IVD from being under a truly free‐swelling state even in a microgravity environment, studies have shown that the height and spinal length of astronauts do increase during missions to the International Space Station (ISS). This is likely due to a combination of both the straightening of the spinal column, and an increase in disc height due to the lower spinal loading compared to on Earth,^20^, ^21^ and increases in IVD height have been reported from best rest studies where participants will have similar low‐magnitude spinal loading over a period of weeks or months.^22^, ^23^ A microgravity environment may also allow astronauts to move around the ISS without substantial ranges of motion being imposed on the spinal column compared to completing daily activities on Earth, as they are able to spin their entire body and propel themselves via guides/rails or freely floating, with the spine maintained in a relatively neutral position. The detrimental effects of a microgravity environment on the musculoskeletal system including a reduction in bone density, and muscle atrophy are well‐reported following space missions, and in order to reduce these effects, astronauts may take pharmacological treatments such as bisphosphonates, and complete short periods of exercise most days during a mission, which may include running with an elasticated harness to simulate partial gravity, cycling, and resistance exercises.^24^, ^25^

This leads to the question posed in the title of this perspective; a large number of whole‐organ IVD culture studies have used relatively low‐magnitude loading and/or no bending of the IVD, which we believe may more closely equate to the loading and kinematics of a microgravity environment than of that on Earth. Additionally, the short‐duration daily periods of higher magnitude loading of some studies (Table 1) may be akin to the loading in a microgravity environment combined with the short‐duration daily exercise regime of astronauts on the ISS. This may offer profound insight into the mechanobiology of the IVD, including the adaptability and resilience of human physiology to low‐gravity and microgravity environments relevant to longer space missions and human settlements on the Moon and Mars, and may have valuable implications for the design of rehabilitation protocols and assistive technologies to mitigate the adverse effects of prolonged exposure to such environments. However, such insights must be contextualized with the larger societal burden of back pain on Earth, which is the focus of the majority of research using bioreactor systems.

## 5 THE FUTURE OF IVD BIOREACTOR RESEARCH

Whole‐organ IVD studies have contributed, and continue to contribute hugely to our understanding of IVD mechanobiology. However, based on the loading regimes used in previous bioreactor studies (Table 1), there is a need to more closely replicate the large range of load magnitudes and rates that IVDs are subjected to in vivo on Earth. This will provide the capability to address the burden of back pain with improved precision and effectiveness. It is also critical to reflect upon the terminology used in studies, and more realistically describe loading conditions with respect to human in vivo loading, recognizing that low‐magnitude and low‐rate loading, and short daily loading periods do not create a physiological loading protocol, even if it may be more physiological than no loading at all.

Previous calls to develop advanced bioreactor systems capable of replicating physiological loading^4^, ^5^, ^6^ are already being recognized, with preliminary studies having been completed to develop test fixtures,^26^ and six‐axis test systems, and loading protocols^27^ for bioreactor studies. These developments provide the potential to replicate the complex loads and kinematics of ADLs. Previous studies using simplified loading regimes have demonstrated the significant effect that the magnitude,^28^ duration,^18^ and rate^29^ of loading can have on cell viability and gene expression. Being able to replicate ADLs will build upon this research by enabling the replication of different populations using bioreactors, including sedentary and active lifestyles, military personnel, individuals involved in contact or extreme sports, specific patient populations, and occupants of the ISS. These capabilities will allow bioreactor studies to provide insight that is not possible through either isolated cell culture or in vivo, animal models,^7^ and the ability to understand how lifestyle factors may impact IVD health will enable the development of effective strategies to minimize the development and progression of IVD degeneration, and provide advanced pre‐clinical testing protocols to evaluate the efficacy of interventions for conditions such as degenerative disc disease.

## CONFLICT OF INTEREST STATEMENT

The authors declare that they have no known competing financial interests or personal relationships that could have appeared to influence the work reported in this paper.
